# How are cell and tissue structure and function influenced by gravity and what are the gravity perception mechanisms?

**DOI:** 10.1038/s41526-024-00357-9

**Published:** 2024-02-10

**Authors:** Trent Davis, Kevin Tabury, Shouan Zhu, Debora Angeloni, Sarah Baatout, Alexandra Benchoua, Juergen Bereiter-Hahn, Daniele Bottai, Judith-Irina Buchheim, Marco Calvaruso, Eugénie Carnero-Diaz, Sara Castiglioni, Duccio Cavalieri, Gabriele Ceccarelli, Alexander Choukér, Francesca Cialdai, Gianni Ciofani, Giuseppe Coppola, Gabriella Cusella, Andrea Degl’Innocenti, Jean-Francois Desaphy, Jean-Pol Frippiat, Michael Gelinsky, Giada Genchi, Maria Grano, Daniela Grimm, Alain Guignandon, Christiane Hahn, Jason Hatton, Raúl Herranz, Christine E. Hellweg, Carlo Saverio Iorio, Thodoris Karapantsios, Jack J.W.A. van Loon, Matteo Lulli, Jeanette Maier, Jos Malda, Emina Mamaca, Lucia Morbidelli, Angelique van Ombergen, Andreas Osterman, Aleksandr Ovsianikov, Francesco Pampaloni, Elizabeth Pavezlorie, Veronica Pereda-Campos, Cyrille Przybyla, Christopher Puhl, Petra Rettberg, Angela Maria Rizzo, Kate Robson-Brown, Leonardo Rossi, Giorgio Russo, Alessandra Salvetti, Daniela Santucci, Matthias Sperl, Sara Tavella, Christiane Thielemann, Ronnie Willaert, Nathaniel Szewczyk, Monica Monici

**Affiliations:** 1 Heritage College of Osteopathic Medicine, Ohio University, Athens, OH USA; 2 Laboratory of Radiobiology, Belgian Nuclear Research Centre, SCK CEN, Mol, Belgium; 3 Institute of Biorobotics, Scuola Superiore Sant’Anna, Pisa, Italy; 4 ISTEM, CECS, AFM-Téléthon, Corbeil-Essonnes, France; 5 Institute for Cell Biology and Neurobiology, Goethe University Frankfurt am Main, Frankfurt am Main, Germany; 6 Department Pharmaceutical Sciences, University of Milan, Milan, Italy; 7 Laboratory of “Translational Research, Stress & Immunity”, Department of Anesthesiology, LMU University Hospital Munich, Munich, Germany; 8 Institute of Molecular Bioimaging and Physiology, National Research Council (IBFM-CNR), Cefalù, Italy; 9 Institute of Systematics, Evolution, Biodiversity, Sorbonne University, NMNH, CNRS, EPHE, UA, Paris, France; 10 Department of Biomedical and Clinical Sciences, University of Milan, Milan, Italy; 11 Department of Biology, University of Florence, Florence, Italy; 12 Department of Public Health, Experimental Medicine and Forensic, University of Pavia, Pavia, Italy; 13 ASAcampus Joint Laboratory, ASA Research Division, DSBSC-University of Florence, Florence, Italy; 14 Smart Bio-Interfaces, Istituto Italiano di Tecnologia, Pontedera, PI 56025 Italy; 15 Institute of Applied Science and Intelligent Systems – CNR, Naples, Italy; 16 Department of Medical Biotechnologies, University of Siena, Italy and Smart Bio-Interfaces, IIT, Pontedera, PI Italy; 17 Department of Precision and Regenerative Medicine, University of Bari “Aldo Moro”, Bari, Italy; 18 Stress, Immunity, Pathogens Laboratory, SIMPA, Université de Lorraine, Nancy, France; 19 Centre for Translational Bone, Joint & Soft Tissue Research, TU Dresden, Dresden, Germany; 20 Department of Microgravity and Translational Regenerative Medicine, Otto-von-Guericke-University Magdeburg, Germany & Dept of Biomedicine, Aarhus University, Aarhus, Denmark; 21 SAINBIOSE, INSERM U1059, Université Jean Monnet, Saint-Etienne, F-42000 France; 22 European Space Agency, ESTEC, Noordwijk, The Netherlands; 23 Centro de Investigaciones Biológicas Margarita Salas (CSIC), Madrid, Spain; 24 Radiation Biology Department, Institute of Aerospace Medicine, German Aerospace Center (DLR), Cologne, Germany; 25 CREST-ATM, Université libre de Bruxelles, Bruxelles, Belgium; 26 Faculty of Chemistry, Aristotle University of Thessaloniki, Thessaloniki, Greece; 27 Amsterdam University Medical Center, ACTA/VU, Amsterdam, Netherlands; 28 Department of Experimental and Clinical Biomedical Sciences, University of Florence, Florence, Italy; 29 Department of Orthopaedics, University Medical Center Utrecht & Department of Clinical Sciences, Utrecht University, Utrecht, The Netherlands; 30 European and International Affairs Department, Ifremer centre Bretagne, Plouzané, France; 31 Department of Life Sciences, University of Siena, Siena, Italy; 32 Max von Pettenkofer Institute, Virology, LMU Munich & DZIF, Partner Site Munich, Munich, Germany; 33 3D Printing and Biofabrication, Institute of Materials Science and Technology, TU Wien, Vienna, Austria; 34 Buchmann Inst. for Molecular Life Sciences, Goethe-Universität Frankfurt am Main, Frankfurt am Main, Germany; 35 Ludwig Boltzmann Institute for Traumatology, Research Center in Cooperation with AUVA, Vienna, Austria; 36 GSBMS/URU EVOLSAN - Medecine Evolutive, Université Paul Sabatier Toulouse III, Toulouse, France; 37 MARBEC, Université de Montpellier, CNRS, Ifremer, IRD, Palavas les Flots, France; 38 Space Applications NV/SA for European Space Agency, Noordwijk, The Netherlands; 39 DLR, Institute of Aerospace Medicine, Research Group Astrobiology, Köln, Germany; 40 Department of Pharmacological and Biomolecular Sciences, University of Milan, Milan, Italy; 41 Department of Engineering Mathematics, and Department of Anthropology and Archaeology, University of Bristol, Bristol, UK; 42 Department of Clinical and Experimental Medicine, University of Pisa, Pisa, Italy; 43 Center for Behavioural Sciences and Mental Health, Istituto Superiore Sanità, Rome, Italy; 44 DLR-MP, Cologne, Germany; 45 IRCCS Ospedale Policlinico San Martino and University of Genoa, DIMES, Genoa, Italy; 46 BioMEMS lab, University of Applied Sciences Aschaffenburg, Aschaffenburg, Germany; 47 Research Group NAMI and NANO, Vrije Universiteit Brussels, Brussels, Belgium

**Keywords:** Cell biology, Systems biology

## Abstract

Progress in mechanobiology allowed us to better understand the important role of mechanical forces in the regulation of biological processes. Space research in the field of life sciences clearly showed that gravity plays a crucial role in biological processes. The space environment offers the unique opportunity to carry out experiments without gravity, helping us not only to understand the effects of gravitational alterations on biological systems but also the mechanisms underlying mechanoperception and cell/tissue response to mechanical and gravitational stresses. Despite the progress made so far, for future space exploration programs it is necessary to increase our knowledge on the mechanotransduction processes as well as on the molecular mechanisms underlying microgravity-induced cell and tissue alterations. This white paper reports the suggestions and recommendations of the SciSpacE Science Community for the elaboration of the section of the European Space Agency roadmap “Biology in Space and Analogue Environments” focusing on “How are cells and tissues influenced by gravity and what are the gravity perception mechanisms?” The knowledge gaps that prevent the Science Community from fully answering this question and the activities proposed to fill them are discussed.

## Introduction

It is well documented that cell behavior is also regulated by mechanical cues the cells receive from their microenvironment^1,2^. Cells perceive changes in the mechanical properties of their environment and alterations in gravity. This perception leads to the elaboration of a biological response which can affect processes at the cell and tissue level. Hence, mechanical and gravitational forces can modulate several functions such as proliferation^3^, differentiation^4^, apoptosis^5–7^, gene expression^8^, signaling^9^, and adhesion/migration properties^10,11^, thus affecting the organization and functioning of 3D structures formed by cells (constructs, tissues, and organs)^12^. The resulting effects might underlie the pathophysiological changes observed at the whole organism level during spaceflight. Therefore, understanding the mechanotransduction processes and the resulting changes, not only in cell functions but also in the extracellular environment (e.g. cell-to-cell communication, extracellular matrix (ECM) production, ECM properties, etc.), is a necessary condition to increase our knowledge on the spaceflight adaptation processes, focusing on mechanobiology.

Over the past two decades, many studies have revealed the crucial role that mechanical stressors of the cell microenvironment play in the control of cell morphology and volume, cell cycle and growth. These studies produced significant progress in the field of mechanobiology. For example, it has been revealed that the stiffness of the microenvironment has an important role in regulating cell differentiation/dedifferentiation^13^, adhesion/migration^14^, and also the onset and progression of serious diseases, such as cancer^15^ and fibrosis^16,17^. Other studies have demonstrated that the process by which cells translate mechanical stimuli into biochemical signals involves cytoskeleton^18^, integrins^19^, ECM molecules^20^, ion channels^21,22^, guanosine triphosphate hydrolase enzymes (GTPases)^23^, and mitogen-activated protein kinases (MAPKs)^24^. However, the exact mechanisms that underlie how cells perceive mechanical stimuli through the diverse mechanosensitive molecules are still not fully understood. Research carried out in altered gravity conditions represents an extraordinary opportunity to investigate the impact of gravity and mechanical forces on biological processes as well as the response of cells and tissues to mechanical and gravitational stimuli.

This white paper reports the suggestions and recommendations of the SciSpacE Science Community (SSC) for answering the question “How are cells and tissues influenced by gravity and what are the gravity perception mechanisms?”, which is one of the major issues of the European Space Agency (ESA) roadmap “Biology in Space and analogue environments”. Key knowledge gaps are indicated and activities to fill them are discussed.

## Key knowledge gaps

**Fig. 1:**
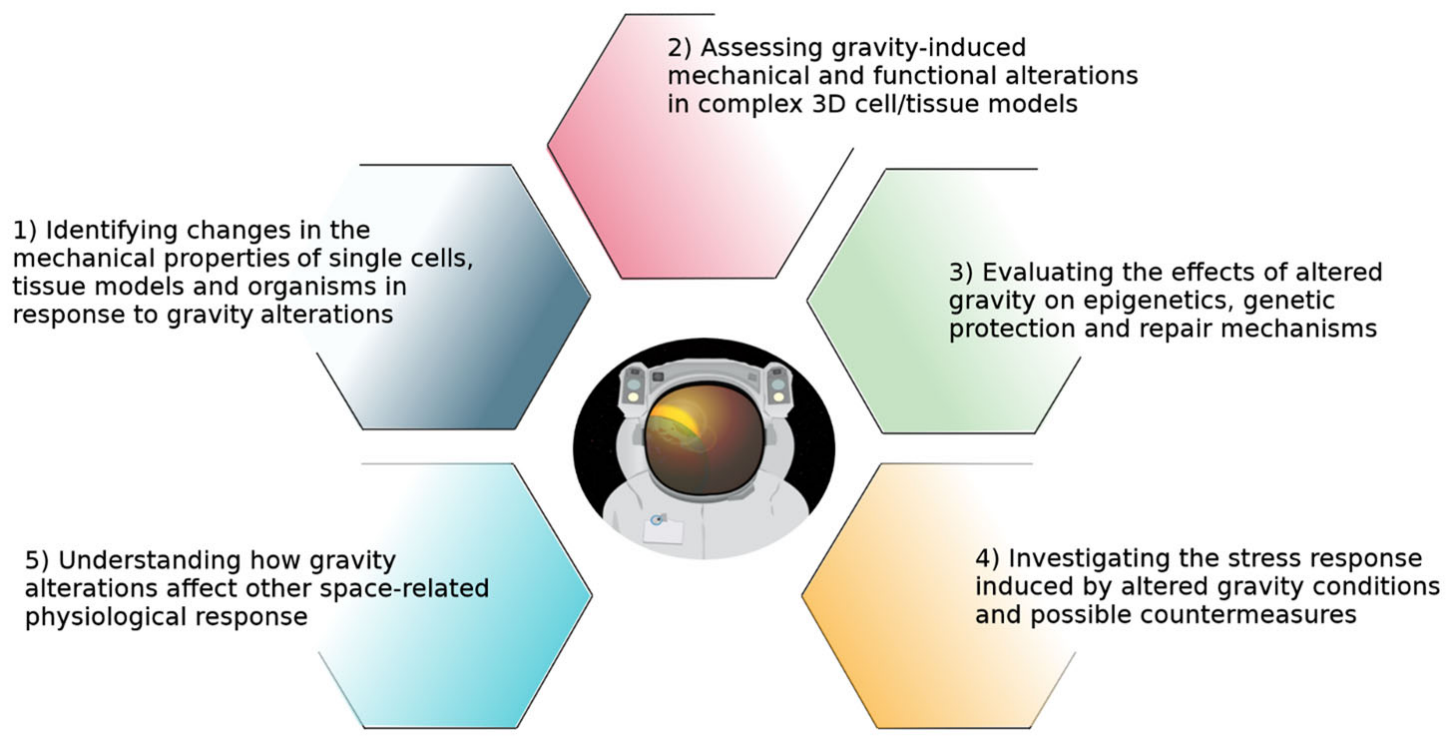
How to fill knowledge gaps. Key issues of great importance to better understand how gravity affects cell and tissue structure and what are the gravity perception mechanisms.

With the avenue of the Artemis program that intends to construct a human-operated Moon base camp in preparation for human space exploration missions of greater distance and duration, five key issues were determined to be of great importance to better understand how gravity affects cell and tissue structure and what are the gravity perception mechanisms (Fig. 1):

1. Identifying changes in the mechanical properties of single cells, tissue models, and organisms in response to gravity alterations.
2. Assessing gravity-induced mechanical and functional alterations in complex 3D cell/tissue models.
3. Evaluating the effects of altered gravity on epigenetics, genetic protection, and repair mechanisms.
4. Investigating the stress response induced by altered gravity conditions and possible countermeasures.
5. Understanding how gravity alterations affect other space-related physiological responses.

In addition, a particular emphasis on the use of complex 3D models was given as these models are crucial to bridge the knowledge of biological processes at the molecular, cellular, and tissue level with human research. In particular, these 3D models are a valuable tool in the efforts to better understand: (i) organ and tissue mechanical properties; (ii) the role of gravity in modulating organ morphology and function; (iii) mechanisms of adaptation to altered gravity, consequent long-term alterations, and possible countermeasures^25,26^. In parallel, cell-based 2D models can also support the elucidation of this understanding.

## Proposed research activities to answer the key knowledge gaps

A fundamental question in space physiology, space medicine, and space biology is how cells adapt to gravity changes. All species that have evolved on Earth possess the structure and function that is adjusted to their living environment, thus to Earth’s gravity. Yet, during spaceflights, astronauts are exposed to microgravity, partial gravity (on the Moon and Mars), and hypergravity. Understanding the mechano-biological coupling mechanisms through which gravity regulates biological responses at molecular, sub-cellular, cellular, tissue, and organism levels is therefore vital. With the aim to reproduce fundamental cell-cell contacts, intra- and inter-cellular signaling, elucidating the underlying mechanisms of how a cell detects, transmits, transduces, and reacts to modified gravitational impulses would aid in identifying changes in the mechanical properties of single cells, tissue models and organisms in response to gravitational alterations.

While, at this stage, it is impossible to recreate the complete physiological complexity of an organ, 3D models (including spheroids, organoids, organ-on-chip and ex-vivo tissue explants from the skin, cornea, retina, blood vessel, muscle tissues, brain, or spinal cord) make it possible to explore tissue-like responses to gravity alterations at the level of morphology, structure, gene/protein expression, cell function, and physiology^27–30^. Therefore, implementing 3D model systems enables the assessment of gravity-induced mechanical and functional alterations at a more complex and higher organizational level. In particular, the understanding of the effects of changes in the transport coefficient of culture fluids on the growth and development of tissues and 3D constructs^31,32^. These 3D models can be used to evaluate or develop new therapeutic treatments. Changes in mitochondrial activity and energy metabolism in response to gravity alterations are also important to investigate. In addition, the comparison between 2D and 3D models would be useful to understand how gravity affects intercellular communication and exchange (proteins, lipids, genetic material, role of extracellular vesicles). Further research is needed about the influence of gravity on the regulation of cell cycle and cell death (in particular cell senescence and apoptosis). Furthermore, studying nanomaterial interactions with biological systems (2D and 3D) in altered gravity conditions at any level (molecular, transcriptional, translational, and phenotypic amongst others) may offer opportunities for developing effective countermeasures (including medications carrying physicochemical cues) against the deleterious effects of the space environment.

Another important aspect to consider when studying the biological effects of microgravity is its interplay with space radiation that poses a constant threat to the DNA integrity of astronauts^33,34^. Cells have developed sophisticated systems that can find and repair DNA lesions as a defense mechanism against the effects of DNA damage. These mechanisms are comprised of a network of cellular proteins that are active participants in the DNA damage response (DDR) pathways. Some examples of these pathways include cell cycle regulation, DNA repair, and apoptosis. In general, cells are able to compensate for moderate DNA damage through these many repair processes. However, the circumstances of space, particularly gravity alterations, may have a negative impact on the DDR pathways, leading to a loss of genomic integrity. Hence, evaluating the effects of altered gravity in combination with space radiation on epigenetics, genetic protection, and repair mechanisms remains crucial.

Furthermore, cells respond to stress through the activation of processes that support cellular functioning and, as a result, the maintenance of micro-environmental and organismal homeostasis^35^. Microgravity is a stressor that disrupts cell homeostasis, which was especially observed in the musculoskeletal, cardiopulmonary, and central nervous system^36–38^. Yet, little is known about the signals that emerge from altered gravity-stressed cells in order to provide a coordinated adaptive response across tissues, organs, and the entire organism. Investigating the stress response induced by altered gravity (from microgravity to hypergravity) remains important to establish proper countermeasures. Therefore, well-designed ground-based research programs that investigate the influence of gravity should accompany in-flight research in order to favor a deeper comprehension of the findings from the in-flight research. Finally, the subcellular and cellular mechanisms related to the adaptation responses (intra- and extra-cellular) to gravity alterations from 2D and 3D models should be the basis for designing in-flight studies to understand space-related physiological changes at the tissue and/or whole organism level. These studies should be conducted in order to gain a better understanding of how space affects the body as a whole.

All the available platforms (International Space Station (ISS), parabolic flights, centrifuges, on-ground systems for modeling micro- or hypogravity, etc.) can be profitably used to test responses of in vitro, ex vivo, and in vivo models to gravitational alterations and the mechanisms of gravity perception. The use of on-ground facilities is helpful to increase the number of tests and better prepare the in-flight experiments. Finally, it is very important to have access to facilities that simultaneously model the different space stressors (for example, microgravity and radiation), to study the resulting combination of effects.

Early outcomes of the activities above described can be obtained in a relatively short time (about 3 years), the implementation of most parts of the activities requires from medium to long time (6–10 years).

## Priorities for the ESA Space Exploration Program

To prevent the onset of diseases, to prepare effective countermeasures, and to develop tools for diagnostics and health monitoring, it is crucial to understand the molecular and cellular mechanisms underlying the processes of adaptation to spaceflight, the resulting pathophysiological changes, and the associated health risks^39–41^. Therefore, the above-indicated activities can be regarded as fundamental support to the future Programs of Space Exploration which concern the stay of humans in space. Specific recommendations for these activities are presented in Table 1.

**Table 1:** Recommendations in short (3 years), middle (6 years), and long term (>10 years)

Open fundamental scientific question	Proposed research activities including ground & space experiments	Suitable testbed environment (Ground, LEO, BLEO, Moon, Mars,)	Space relevance (importance of altered gravity and/or relevance for space exploration)	Timeline (short, medium, long)
1. Identifying changes in the mechanical properties of single cells, tissue models and organisms in response to gravity alterations	Clarify the mechano-biological coupling mechanisms through which gravity regulates biological responses at biopolymers, sub-cellular, cellular, tissue and organism levels with the aim to reproduce fundamental cell–cell contacts, intra- and inter-cellular signaling.	Ground-based (micro- and partial gravity simulation and hyper-gravity) in vitro and in vivo models in/beyond LEO Humans in LEO Humans beyond LEO	Use of spaceflight environment for basic research: Understanding the role of gravity in biological systems Extended microgravity to permit the effect of sub 1×*g* accelerations to be explored Ground-based studies can explore response in microgravity analog or hypergravity	Short - Medium
2. Assessing gravity-induced mechanical and functional alterations in complex 3D cell/tissue models.	Implementing 3D model systems for biological studies in altered gravity conditions: 3D cell cultures (spheroids, organoids, 3D bioprinter, organ-on-a-chip, organotypic cultures) and ex vivo tissue cultures (e.g. skin, cornea, eye lens, retina, blood vessel, muscle tissues, brain slices, spinal cord slices, etc.) with the aim to reproduce fundamental cell-cell contacts, inter-cellular signaling, and mechanical properties of organs (e.g. liver, pancreas, gut, brain). The full physiological complexity of an organ cannot be recapitulated, yet specific organ compartments and functions become experimentally accessible with 3D models, which allow studying the tissue-like response to altered gravity at levels of morphology and structure, gene and protein expression, cell function, and physiological processes. 3D cell cultures and ex vivo tissue cultures can be used also as models of injuries and diseases and to evaluate or develop new therapeutic treatments. Understanding the effects of changes in the transport coefficient of culture fluids on the growth and development of tissues and 3D constructs. Understanding the role of functional changes in mitochondrial and energy metabolism in the overall response to spaceflight. (*)Studying how gravity determines intercellular communication and exchange (proteins, lipids, genetic material, role of extracellular vesicles (EVs)), the regulation of cell cycle and death (in particular cell senescence and apoptosis), and consequent protective and deleterious effects in 2D and 3D in vitro models. (*)Identify the cellular and sub-cellular processes that underlie the effects of altered gravity (micro-, partial, one, and hypergravity) seen in 2D and 3D in vitro models, ex-vivo explants, and higher tissue and organism organizations. (*)Studying nanomaterial interaction with biological systems in altered gravity conditions at any level (molecular, transcriptional, translational, phenotypic, etc.). The deriving knowledge may offer opportunities for developing effective countermeasures (including medications carrying physicochemical cues) against deleterious effects of the space environment. (*)Note: These statements are very true both for 2D and 3D systems and the comparison between 2D and 3D systems is very interesting. Therefore, these activities should take place for questions 1 and 2.	Ground-based (micro- and partial gravity simulation and hyper-gravity) in vitro and ex vivo models in/beyond LEO	Use of spaceflight environment for basic and applied research: Extended microgravity to permit the effect of sub 1×*g* accelerations to be explored Ground based studies can explore response in micro- and partial gravity analog and hypergravity Microgravity conditions may facilitate assembly of 3D cell constructions and bioprinting due to lack of sedimentation Space Exploration relevance: Understanding effect of spaceflight on human tissues and organ systems & development of countermeasures. 3D cell cultures provide in-vitro models to understand how tissue/organ function is altered by spaceflight & potentially explore therapeutic / countermeasures	Medium
3. Evaluating the effects of altered gravity on epigenetics, genetic protection, and repair mechanisms.	Analyzing the activation of the DNA Damage Response (DDR) which encompasses e.g. DNA repair, cell cycle arrest, possibly cell death, and gene expression changes after 2D/3D cell cultures exposure to real and simulated microgravity, also in association with “in vitro” exposure to high energy particle radiation and countermeasures.	Ground-based (micro- and partial gravity simulation and hyper-gravity) in combination with radiation (e.g. clinostat at heavy ion accelerator and other altered gravity simulation systems coupled with radiation sources) in vitro and in vivo models in/beyond LEO Humans in LEO Humans beyond LEO	Use of spaceflight environment for basic and applied research: Extended microgravity to permit the effect of sub 1×*g* accelerations to be explored on processes Interaction between microgravity and cosmic radiation effects	Short Medium
4. Investigating the stress response induced by altered gravity conditions and possible countermeasures.	In-flight studies should be accompanied by a well-designed ground-based research program exploring the impact of gravity to better understand the in-flight results. Studies should explore the gravity continuum (from microgravity to hypergravity) of their in vitro and/or in vivo systems	Ground-based (micro- and partial gravity simulation and hyper-gravity) in vitro and in vivo models in/beyond LEO Humans in LEO Humans beyond LEO	Use of spaceflight environment for basic and applied research: Extended microgravity to permit the effect of sub 1×*g* accelerations to be explored Ground-based studies can explore response in microgravity analog or hypergravity.	Short Medium
5. Understanding how gravitational alterations affect other space-related physiological responses.	In-flight studies should be designed to link mechanisms identified in question 1 to space-related physiological changes at the tissue and/or whole organism level.	Ground-based (micro- and partial gravity simulation and hyper-gravity) in vitro and in vivo models in/beyond LEO Humans in LEO Humans beyond LEO	Use of spaceflight environment for basic and applied research: Linking basic gravity sensing mechanisms to physiological alterations. Applying knowledge of basic gravity sensing mechanisms regulating physiological alterations to applied countermeasures. Space Exploration relevance: Understanding the effect of spaceflight on human tissues and organ systems & development of countermeasures.	Medium Long

## Benefit for Earth and industrial relevance

The conditions encountered during spaceflights, especially microgravity, offer a unique opportunity to eliminate the stress caused by mechanical forces from a living system over the long-term, regardless of whether the system in question is a cell, a tissue, or an entire organism. Hence, such an environment provides optimal research conditions to measure the impact of gravity and mechanical forces on biological processes and living systems^42^. By taking advantage of this one-of-a-kind research environment, we have the chance to advance our fundamental understanding of the cellular mechanisms that are involved in gravity or mechano-sensing, as well as the response that is induced by them. Beside supporting space exploration missions, these findings have the potential to find applications on Earth in many different fields, such as general medicine, rehabilitation, and training, just to mention some examples. Indeed, in the biomedical field, the knowledge gained through space research can provide huge translational value for understanding disease conditions, provide novel therapeutic targets, and, consequently, new therapeutic strategies as well as personalized treatments^43^. Further information on the role of gravitational/mechanical stress in biological processes can bring progress not only in the biomedicine area, but also in the fields of physiatry, rehabilitation, sports medicine, and training. The advancement of technology is one of the key benefits of space exploration. For example, remote sensing technologies could be useful in the study of the epidemiology of infectious diseases. Data may be used to monitor disease patterns, understand environmental triggers for the spread of diseases, predict risk areas, and define regions that require disease-control planning^44,45^. This is particularly important in developing countries, where infectious diseases remain among the top causes of death. Space exploration also offers the potential for new discoveries in pharmacology. Drugs widely used on Earth, such as bisphosphonates, have been tested in Space^46^. Moreover, during spaceflight, humans live in conditions of isolation and confinement, in an environment where air, water, food, and waste are recycled. Hence, the risk of pathogen development may increase. This condition, associated with alterations in astronauts’ microbial flora and possible decrease in drug susceptibility, due to possible changes in physical–chemical properties of drugs as well as to changes in drug pharmacodynamics and pharmacokinetics, makes Space an excellent environmental model to study and develop new drugs^47,48^.

Finally, the findings obtained from space exploration missions can be directly used in the R&D process for several industries including manufacturing, materials, cosmetics, and many more.

## Discussion

From the perspective of future long-duration missions in/beyond the Low Earth Orbit (LEO), it is imperative to expand our knowledge in the fields of space biology and physiology. In this chapter of the SSC white paper, we indicated the activities needed to improve our understanding of how gravity influences biological processes as well as cell and tissue structure and function in biological systems. A very important topic is the investigation of the gravity perception mechanisms, which are still far from clear and whose comprehension is crucial to identify the pathways that transduce the gravitational alterations in biological response. Table 1 summarizes the main questions still unresolved, the activities aimed at answering these scientific problems, the platforms and timelines necessary to conduct the above research, and the repercussions that the results could have both on future space exploration programs and in the biotechnological and biomedical fields on Earth.

### Reporting summary

Further information on research design is available in the Nature Research Reporting Summary linked to this article.

### Supplementary information

Reporting Summary

## References

[1] Bradbury P (2020). Modeling the impact of microgravity at the cellular level: implications for human disease. Front. Cell Dev. Biol..

[2] Vandenburgh HH (1992). Mechanical forces and their second messengers in stimulating cell growth in vitro. Am. J. Physiol..

[3] Unsworth BR, Lelkes PI (1998). Growing tissues in microgravity. Nat. Med..

[4] Imura T, Otsuka T, Kawahara Y, Yuge L (2019). “Microgravity” as a unique and useful stem cell culture environment for cell-based therapy. Regen. Ther..

[5] Morbidelli L (2005). Simulated hypogravity impairs the angiogenic response of endothelium by up-regulating apoptotic signals. Biochem. Biophys. Res. Commun..

[6] Monici M (2006). Modeled gravitational unloading triggers differentiation and apoptosis in preosteoclastic cells. J. Cell. Biochem..

[7] Riwaldt S (2021). Role of apoptosis in wound healing and apoptosis alterations in microgravity. Front. Bioeng. Biotechnol..

[8] Panciera T, Azzolin L, Cordenonsi M, Piccolo S (2017). Mechanobiology of YAP and TAZ in physiology and disease. Nat. Rev. Mol. Cell Biol..

[9] Siamwala JH, Rajendran S, Chatterjee S (2015). Strategies of manipulating BMP signaling in microgravity to prevent bone loss. Vitam. Horm..

[10] Maier JA, Cialdai F, Monici M, Morbidelli L (2015). The impact of microgravity and hypergravity on endothelial cells. Biomed. Res. Int..

[11] Cialdai F (2017). Modeled microgravity affects fibroblast functions related to wound healing. Microgravity Sci. Technol..

[12] Zhang C, Li L, Chen J, Wang J (2015). Behavior of stem cells under outer-space microgravity and ground-based microgravity simulation. Cell Biol. Int..

[13] Humphrey JD, Dufresne ER, Schwartz MA (2014). Mechanotransduction and extracellular matrix homeostasis. Nat. Rev. Mol. Cell Biol..

[14] Doyle AD, Yamada KM (2016). Mechanosensing via cell-matrix adhesions in 3D microenvironments. Exp. Cell Res..

[15] Grimm D (2022). The fight against cancer by microgravity: the multicellular spheroid as a metastasis model. Int. J. Mol. Sci..

[16] Piersma B, Hayward MK, Weaver VM (2020). Fibrosis and cancer: a strained relationship. Biochim. Biophys. Acta Rev. Cancer.

[17] Cialdai F, Risaliti C, Monici M (2022). Role of fibroblasts in wound healing and tissue remodeling on Earth and in space. Front. Bioeng. Biotechnol..

[18] Vorselen D (2014). The role of the cytoskeleton in sensing changes in gravity by nonspecialized cells. FASEB J..

[19] Zhong J, Yang Y, Liao L, Zhang C (2020). Matrix stiffness-regulated cellular functions under different dimensionalities. Biomater. Sci..

[20] Buravkova L, Larina I, Andreeva E, Grigoriev A (2021). Microgravity effects on the matrisome. Cells.

[21] Nday CM, Frantzidis C, Jackson G, Bamidis P, Kourtidou-Papadeli C (2019). Neurophysiological changes in simulated microgravity: an animal model. Neurol. India.

[22] Locatelli L, Maier JAM (2021). Cytoskeletal remodeling mimics endothelial response to microgravity. Front. Cell Dev. Biol..

[23] Louis F, Deroanne C, Nusgens B, Vico L, Guignandon A (2015). RhoGTPases as key players in mammalian cell adaptation to microgravity. Biomed. Res. Int..

[24] Sun Y, Kuang Y, Zuo Z (2021). The emerging role of macrophages in immune system dysfunction under real and simulated microgravity conditions. Int. J. Mol. Sci..

[25] Navran S (2008). The application of low shear modeled microgravity to 3-D cell biology and tissue engineering. Biotechnol. Annu. Rev..

[26] Aleshcheva G (2016). Scaffold-free tissue formation under real and simulated microgravity conditions. Basic Clin. Pharm. Toxicol..

[27] Jensen C, Teng Y (2020). Is it time to start transitioning from 2D to 3D cell culture?. Front. Mol. Biosci..

[28] Moroni L (2022). What can biofabrication do for space and what can space do for biofabrication?. Trends Biotechnol..

[29] Edmondson R, Broglie JJ, Adcock AF, Yang L (2014). Three-dimensional cell culture systems and their applications in drug discovery and cell-based biosensors. ASSAY Drug Dev. Technol..

[30] Cubo-Mateo N (2020). Can 3D bioprinting be a key for exploratory missions and human settlements on the Moon and Mars?. Biofabrication.

[31] Mekala NK, Baadhe RR, Potumarthi R (2014). Mass transfer aspects of 3D cell cultures in tissue engineering. Asia-Pac. J. Chem. Eng..

[32] Nijhuis J, Schmidt S, Tran NN, Hessel V (2022). Microfluidics and macrofluidics in space: ISS-proven fluidic transport and handling concepts. Front. Space Technol..

[33] Moreno-Villanueva M (2017). Interplay of space radiation and microgravity in DNA damage and DNA damage response. NPJ Microgravity.

[34] Barravecchia I (2021). Microgravity and space radiation inhibit autophagy in human capillary endothelial cells, through either opposite or synergistic effects on specific molecular pathways. Cell. Mol. Life Sci..

[35] Galluzzi L, Yamazaki T, Kroemer G (2018). Linking cellular stress responses to systemic homeostasis. Nat. Rev. Mol. Cell Biol..

[36] Juhl OJ (2021). Update on the effects of microgravity on the musculoskeletal system. NPJ Microgravity.

[37] Baran R (2021). The cardiovascular system in space: focus on in vivo and in vitro studies. Biomedicines.

[38] Clément GR (2020). Challenges to the central nervous system during human spaceflight missions to Mars. J. Neurophysiol..

[39] Steller JG (2018). Oxidative stress as cause, consequence, or biomarker of altered female reproduction and development in the space environment. Int. J. Mol. Sci..

[40] Zhang Y (2017). Transcriptomics, NF-κB pathway, and their potential spaceflight-related health consequences. Int. J. Mol. Sci..

[41] Johnson I, Nguyen CT, Wise P, Grimm D (2020). Implications of altered endosome and lysosome biology in space environments. Int. J. Mol. Sci..

[42] Davis SA, Davis BL (2012). Exercise equipment used in microgravity: challenges and opportunities. Curr. Sports Med. Rep..

[43] Pavez Loriè E (2021). The future of personalized medicine in space: from observations to countermeasures. Front. Bioeng. Biotechnol..

[44] Beck LR, Lobitz BM, Wood BL (2000). Remote sensing and human health: new sensors and new opportunities. Emerg. Infect. Dis..

[45] De La Rocque S, Michel V, Plazanet D, Pin R (2004). Remote sensing and epidemiology: examples of applications for two vector-borne diseases. Comp. Immunol. Microbiol. Infect. Dis..

[46] Leblanc A (2013). Bisphosphonates as a supplement to exercise to protect bone during long-duration spaceflight. Osteoporos. Int..

[47] Braddock M (2020). From target identification to drug development in space: using the microgravity assist. Curr. Drug Discov. Technol..

[48] Dello Russo C (2022). Physiological adaptations affecting drug pharmacokinetics in space: what do we really know? A critical review of the literature. Br. J. Pharmacol..

